# Growth and antioxidant status of broilers fed supplemental lysine and pyridoxine under high ambient temperature

**Published:** 2015-06-15

**Authors:** Farzaneh Khakpour Irani, Mohsen Daneshyar, Ramin Najafi

**Affiliations:** *Department of Animal Science, Faculty of Agriculture, Urmia University, Urmia, Iran.*

**Keywords:** Blood indices, Glutathione peroxidase, Malondialdehyde, Superoxide dismutase

## Abstract

Three levels of lysine (90, 100 and 110% of Ross requirement) and of pyridoxine (3, 6 and 9 mg kg^-1^) were used in a 3 × 3 factorial experiment to investigate the growth and blood antioxidant ability of broilers under high ambient temperature. None of the dietary supplements affected the weight gain during the starter and grower periods. Although no significant differences were detected between the treatments during the entire period, high lysine level fed birds had a lower weight gain. At any levels of pyridoxine, high lysine fed birds were lighter than others. Neither the lysine nor pyridoxine changed the feed intake or feed conversion ratio during the starter, grower and entire period. However there was no significant difference between the treatments for blood malondialdehyde (MDA) concentration, medium lysine fed birds had lower blood MDA than other ones. No significant effects on blood triglyceride, total protein and blood superoxide dismutase activity were indicated with addition of any lysine or pyridoxine level. Medium lysine fed birds had decreased blood glutathione peroxidase activity compared to the birds of other treatments. It was concluded that providing the proposed dietary lysine requirement of Ross strain during heat stress ensuring the best body weight gain and body antioxidant ability. Higher lysine level causes the retarded weight gain due to higher excretion of arginine from the body and consequently higher lipid peroxidation.

## Introduction

Thermal stress exerts its deleterious effects on performance, carcass yield, mortality rates and morbidity in poultry. It results in the generation of enormous reactive oxygen species (ROS) that overwhelms tissue antioxidant defense. Antioxidant supplementation provides beneficial effects against stress-induced tissue damage.^1^ Lysine is the second limiting amino acid.^2^ The lysine deficiency increases wrap restraint stress induced fecal excretion of lysine and stress-induced anxiety.^3^ Han and Baker reported specifically increased lysine requirements during acute stress in broilers.^4^ Pyridoxine is associated against lipid peroxidation.^5^ Marginal pyridoxine contents increased lipid peroxidation and considerably stimulated the activity of glutathione-dependent enzyme.^6^ Pyridoxine supplementation prevented the oxidative stress and reduced lipid peroxidation in rat.^7^ The present study was aimed at investigate the effect of dietary lysine and pyridoxine on broilers exposed to the heat stress.

## Materials and Methods

**Birds and housing. **Four hundred and fifty newly hatched broiler chicks (Ross 308) were used in a 3 × 3 factorial design based on a completely randomized design. The birds were randomly assigned to nine treatment groups consisting of five replicates and 10 birds each. Continuous temperature of 32 ± 1 ˚C was used in the house for inducing heat stress throughout the experimental period (day 1 to 42 of age). The birds were fed a starter diet until day 21 of age and a grower diet afterwards (from day 22 to 42 of age), (Table 1). The experimental diets were produced after grinding (for the ingredients such as corn, soybean meal and wheat) by a hammer mill and mixing by a horizontal mixer. The diet and water were given *ad libitum*. Twenty-three hours of light was provided throughout the experiment. Three lysine (Paik Kwang Industrial Co. Ltd., Kunsan City, South Korea) levels of 90% (1.29 and 1.12%), 100% (1.43 and 1.24%), 110% (1.57 and 1.36%) of Ross company requirement were used respectively for starter and grower periods. Moreover, three constant levels of 3, 6 and 9 mg kg^-1^ pyridoxine (Adisseo Co. Ltd., Antony City, France) were fed throughout the experimental period.

**Table 1:** Composition of basal diets.^1^

**Ingredients**	**Starter (%)**	**Grower (%)**
**Corn**	31.6	32.52
**Soybean meal**	39.57	33.84
**Soybean oil**	3.9	4.33
**Wheat**	20	25
**Dicalcium phosphate**	2.10	2.15
**Limestone**	1.1	0.86
**DL-Methionine**	0.38	0.08
**Vitamin/Mineral premix**	0.5	0.5
**Lysine HCL**	0.11	0.06
**Salt**	0.37	0.34
**Sand**	0.37	0.32
**Total**	100	100
***Calculated composition***		
**Metabolizable energy (Kcal kg** ^-1^ **)**	2900	3000
**Crude protein (%)**	21.99	20.00
**Lysine (%)**	1.29	1.12
**Methionine (%)**	0.70	0.38
**Methionine + Cysteine (%)**	1.07	0.73
**Arginine (%)**	1.53	1.38
**Tryptophan (%)**	0.29	0.26
**Calcium (%)**	1.00	0.90
**Available phosphorus (%)**	0.45	0.45
**Pyridoxine (mg kg** ^-1^ **)**	3.00	3.00

1 supplied per kg of diet contained: retinol, 9000 IU; chole-calciferol 2000 IU; Alpha-tocopherol 18 IU; Menadione 2 mg, Thiamin 1.8 mg; Riboflavin 6.6 mg; Niacin 30 mg; Pyridoxine 3 mg; Folic acid 1 mg; Vitamin B_12_ 0.015 mg; Biotin 0.1 mg; Choline chloride 500 mg; Ethoxyquin 100 mg; Manganese 100 mg; Iron 50 mg; Zinc 85 mg; Copper 10 mg; Iodine 1 mg; and Selenium 0.2 mg.

**Growth performance.** Body weight gain (BWG), feed intake (FI) and FCR were determined during the starter, grower and whole the experimental periods. Furthermore, total mortality of each pen at the end of the experiment was divided by the number of birds at the beginning of the experiment (day 1), and transformed by arc sin √y.^8^

**Blood parameters.** At the end of experiment (day 42 of age), one bird per replicate (five per treatment) was selected randomly and slaughtered. Two series of blood sample were collected in anticoagulant tubes at slaughter.

Then one series of blood samples were transferred to the laboratory and their plasma were separated by centrifuging at 5,000 rpm for 5 min. Then these plasma samples were stored in – 20 ˚Calong with other series of blood samples until the later analyses. The activity of blood superoxide dismutase (SOD) and glutathione peroxidase (GPX) enzymes were determined spectro-photometrically by Ransod and Ransel kit (RANDOX Laboratories Ltd., London, UK), respectively. Plasma malondialdehyde (MDA) concentration was measured using MDA reaction with thiobarbituric acid followed by extraction with n-butanol (Merck Co., Darmstadt, Germany). Plasma total protein and tri-glyceride was determined using a spectrophotometer by commercial kits (Pars Azmoon Co., Tehran, Iran).

**Statistical analyses.** A 3×3 factorial experiment was performed with dietary lysine (90, 100 and 110 % of Ross strain requirements) and B6 (3, 6 and 9 mg kg^-1^) as the main factors with five replicates per treatment. The data were analyzed by analysis of variance and using the general liner model procedure of SAS (Version 9.2; SAS Institute, Carry, USA). Pyridoxine and lysine were the variables and their interaction was compared by two way ANOVA. If a significant model (*p* < 0.05) was discerned, treatment means were compared by Tukey’s multiple comparison. The experimental protocols were reviewed and approved by the Animal Care Committee of Urmia University.

## Results

Effect of lysine and pyridoxine supplementation on BWG is presented in Table 2. Neither the lysine nor pyridoxine affected the BWG during the starter and grower periods (*p* > 0.05). However, there were no significant differences between the treatments during whole the experimental period, high lysine level (110% of Ross strain requirement) fed birds had a lower BWG than others (*p* > 0.05). At any levels of pyridoxine, high lysine level fed broiler were lighter than others (*p* < 0.05). None of the dietary lysine or pyridoxine changed the FI or FCR during the starter, grower and whole the experimental periods (unpresented data), (*p* > 0.05).

**Table 2:** Effect of different dietary levels of lysine (L) requirements including 90(L1), 100 (L2) and 110% (L3) of Ross strain and 3(B1), 6 (B2) and 9 (B3) mg kg^-1^ vitamin B_6_ (B) on body weight gain of broiler chickens during the starter (day 1 to 21 of age), grower (day 22 to 42 of age) and whole the experimental (day 1 to 42 of age) periods exposed to a high ambient temperature (32 ± 1 ˚C).

**Lysine**	**Vitamin B**_6_	**Starter**	**Grower**	**Total**
**L1**	**B1**	689.50	1378.34	2071.34
**L1**	**B2**	700.20	1306.36	2017.36
**L1**	**B3**	700.00	1324.32	2016.95
**L2**	**B1**	680.00	1322.00	2002.00
**L2**	**B2**	673.80	1434.17	2124.05
**L2**	**B3**	718.50	1359.25	2076.62
**L3**	**B1**	644.80	1264.03	1908.83
**L3**	**B2**	651.80	1231.53	1929.78
**L3**	**B3**	715.00	1255.00	1951.83
**Pooled SEM**		9.99	20.84	23.40
***p*** **-value**				
**L**		0.54	0.06	0.04
**B**		0.22	0.97	0.81
**L × B**		0.83	0.62	0.78

None of the lysine or pyridoxine affected the mortality during whole the experimental period (unpublished data). Dietary supplementation effects of lysine and pyridoxine on blood antioxidant indices are indicated in Table 3. Lysine supplementation affected the plasma MDA concentration at week 6 of age (*p* < 0.05). There was no significant difference between the treatments for plasma MDA, triglyceride and total protein concentrations. No significant effects on blood SOD activity with addition of any level of lysine or pyridoxine were detected at week 6 of age (*p* > 0.05). Chicks fed medium lysine level (100% Ross strain requirements) had decreased blood GPX activity (*p* < 0.05) as compared to control birds. Furthermore, no effects of pyridoxine or pyridoxine-lysine interaction were detected on performance and blood antioxidant indices in current experiment (Table 3).

**Table 3:** Effect of different dietary levels of lysine (L) requirements including 90 (L1), 100 (L2) and 110% (L3) of Ross strain and 3 (B1), 6 (B2) and 9 (B3) mg kg^-1^ vitamin B_6_ (B) on blood malondialdehyde and activities of glutathione peroxidase, super oxide dismutase, triglyceride and total protein of chickens at week 6 of age exposed to a high ambient temperature (32 ± 1 ˚C).

**Lysine**	**Vitamin B**_6_	**MDA (nmol mL** ^-1^ **)**	**SOD (U g** ^-1^ ** Hb)**	**GPX (U g** ^-1^ ** Hb)**	**Triglyceride (mg dL** ^-1^ **)**	**Total protein (g dL** ^-1^ **)**
**L1**	**B1**	1.32	1403.32	40.43^a^	51.25	4.12
**L1**	**B2**	1.90	1340.55	40.26^ab^	36.00	4.02
**L1**	**B3**	1.98	1206.59	35.55^ab^	74.80	4.08
**L2**	**B1**	1.22	1235.33	34.41^b^	48.25	3.77
**L2**	**B2**	1.28	1239.83	35.41^b^	45.80	4.26
**L2**	**B3**	1.30	1223.56	36.20^ab^	57.80	3.82
**L3**	**B1**	1.82	1304.48	38.96^ab^	50.80	3.98
**L3**	**B2**	2.32	1273.08	39.21^ab^	44.80	4.34
**L3**	**B3**	2.54	1362.58	37.53^ab^	59.80	4.76
**Pooled SEM**		0.10	3.65	0.47	17.71	0.12
***p*** **-value**						
**L**		0.001	0.08	0.004	0.70	0.32
**B**		0.10	0.51	0.14	0.09	0.54
**L × B**		0.67	0.09	0.56	0.96	0.57

## Discussion

The lower growth response of broiler chickens to highest lysine level in our experiment is in consistent with those of Han and Baker.^4^ They suggested that heat stress at 37 ˚C did not increase the requirements of broiler chickens to lysine. Even excessive dietary lysine increases the arginine requirement. Excess dietary lysine increases the requirements of broiler chickens for arginine in great amount which is related to specific antagonism between arginine and lysine. Brake *et al.* concluded that the arginine requirement of hyperthermic birds possibly increased due to an interaction with lysine.^2^ Excessive levels of dietary lysine have resulted in growth depression in poultry which have been alleviated by additional dietary arginine. Hence, lower BWG of highest lysine fed birds is related to higher lysine to arginine ratio which has caused the higher plasma MDA concentration. The arginine is a nutritionally important amino acid and plays multiple physiologic functions in animals. One of these functions is to increase antioxidant ability, reduce superoxide release, and ameliorate lipid peroxidation.^9^ However, increasing extracellular concentrations of arginine regulate the meta-bolism of protein, glucose and lipids in favor of lean tissue gain and white-fat reduction in animals.^10^ Also, arginine metabolizes to nitric oxide, proline, glutamine and poly-amines with enormous biological importance in animals. For example, physiological levels of these metabolites can attenuate the stress response, enhance the immune function, regulate protein synthesis and promote wound healing.^11^ Physiological levels of arginine and nitric oxide have anti-oxidative function.^12^ L-arginine supplementation is hypo-thesized to reduce endothelial dysfunction and athero-genesis via increased biosynthesis of nitric oxide. Also, reduced copper-induced lipid peroxidation has reported by arginine. In an experiment, dietary supplementation of 1.00% arginine increased serum concentrations of proline and glutamine + glutamate and creatine.^13^ It has shown that proline attenuates the stress response in the central nervous system in broiler chickens.^14^

Higher plasma MDA content of highest lysine level fed birds in recent experiment is due to the lower ratio of arginine to lysine which possibly has resulted in higher excretion of arginine from the body. Moreover, consumption of medium lysine level decreased the blood GPX activity in recent experiment. However, we did not investigate the GPX expression, the low activity possibly is related to lower expression of this enzyme due to lower peroxidation. It has been reported that high amounts of peroxidation increases the GPX expression. For example, up regulation of this enzyme has proposed for higher activity of GPX in lung mitochondria of broiler chickens with pulmonary hypertension syndrome connected to its response to greater hydrogen peroxide production.^15^ No effects of pyridoxine or pyridoxine-lysine interaction were detected on performance and blood antioxidant indices in current experiment. Antioxidant effects of pyridoxine have been reported in rats with pyridoxine deficiency. Mahfouz and Kummerow have shown antioxidative effect of pyridoxine supplementation (60 mg kg^-1^) in homo-cysteinemic rats.^7^ Kannan and Jain indicated the inhibition effects of pyridoxine against oxygen radical generation, lipid peroxidation and mitochondrial membrane damage in U937 monocytes.^16^ In the above mentioned experiments, antioxidant effects of pyridoxine have been observed under pyridoxine deficiency. We had no pyridoxine deficiency in current experiment and seem that higher amounts of this vitamin (6 and 9 mg) cannot improve the antioxidant status and performance of birds fed the enough amount of pyridoxine (3 mg kg^-1^).

In conclusion,based on the results of our experiment, providing the proposed dietary lysine requirement of Ross 308 strain (Aviagen Inc.) during heat stress ensuring the best BWG and body antioxidant defense. Higher level of lysine causes the retarded BWG due to higher excretion of arginine from the body and consequently higher lipid peroxidation. Furthermore, decreased blood GPX activity of medium lysine level fed birds possibly is related to lower expression of this enzyme due to lower peroxidation. Furthermore, 3 mg kg^-1 ^pyridoxine is sufficient to support the antioxidant status and performance of the birds and feeding the higher amounts of this vitamin alone or along with lysine has no priority.
